# Exploratory Evaluation of the P22 ELISA for Antibody Detection in Fecal Samples from Tuberculosis-Infected Goat Herds

**DOI:** 10.3390/vetsci13101083

**Published:** 2026-10-13

**Authors:** Javier Ortega, Jose A. Infantes-Lorenzo, Alberto Gómez-Buendía, Álvaro Roy, Carlos Velasco, Beatriz Romero, Inmaculada Moreno, Mercedes Domínguez, Javier Bezos

**Affiliations:** 1 VISAVET Health Surveillance Centre, Complutense University of Madrid, 28040 Madrid, Spain; 2 Reference and Research Laboratory for Respiratory Virus, Centro Nacional de Microbiología, Instituto de Salud Carlos III, Majadahonda, 28040 Madrid, Spain; 3 Departamento de Sanidad Animal, Facultad de Veterinaria, Universidad Complutense de Madrid, 28040 Madrid, Spain; 4 Centro Nacional de Epidemiología, Instituto de Salud Carlos III, 28040 Madrid, Spain; 5 Unidad de Inmunología Microbiana, Centro Nacional de Microbiología, Instituto de Salud Carlos III, Majadahonda, 28040 Madrid, Spain

**Keywords:** caprine tuberculosis, diagnosis, P22 ELISA, fecal samples, antibodies, non-invasive sampling, goats

## Abstract

**Background**: Caprine tuberculosis (cTB), caused by members of the *Mycobacterium tuberculosis* complex (MTBC) represents a significant concern for animal and public health. Antibody-based diagnostic tests have emerged as promising tools to complement ante-mortem official diagnostic techniques in ruminants, which mainly use serum, although alternative, easier-to-collect samples, such as milk, oral fluid, or feces, could also be used. The objective of this exploratory study was to evaluate the reactivity of an in-house P22 enzyme-linked immunosorbent assay (ELISA) in fecal samples from goats belonging to cTB-infected herds, using serum P22 ELISA and official diagnostic tests as comparators. **Methods**: Serum and fecal samples were collected from 51 and 42 adult female goats from a high-prevalence herd (*n* = 149) and a low-prevalence herd (*n* = 220), respectively, which defecated during sampling. These animals were subjected to official diagnostic techniques: single and comparative intradermal tuberculin tests and the interferon-gamma release assay. As no TB-free herd was available, a preliminary fecal cut-off was established by receiver operating characteristic analysis using serum P22 ELISA as the reference classification. **Results**: The percentage of positive animals in fecal samples from the high-prevalence herd was 68.6%, while no positive results were observed in the low-prevalence herd. Serum samples showed a higher number of positives in both herds compared to fecal samples (*p* < 0.05). The agreement between official diagnostic techniques and P22 ELISA (using serum or feces) was slight in general terms. When a combined classification based on all official tests was used as the reference, fecal P22 ELISA detected 58.0% of test-positive goats, none of them in the low-prevalence herd. **Conclusions**: Under the conditions of this field study (no TB-free controls or post-mortem confirmation of infection were available), fecal P22 ELISA showed limited reactivity compared with serum, particularly in the low-prevalence herd, suggesting limited usefulness for antibody detection in TB infected goats.

## Simple Summary

The detection of goats infected with tuberculosis (TB) is challenging, as the current diagnostic tests are not perfect in terms of sensitivity and specificity. Fecal samples can be collected without restraining or stressing the animals and can be obtained from goats of any age and sex, but they have not been evaluated for the detection of antibodies against the agents of TB. In this exploratory study, an in-house antibody test (P22 ELISA) was adapted to fecal samples and applied to adult goats from two TB-infected herds (one with a high and one with a low prevalence of infection), and its reactivity was compared with that obtained by the same test in serum and with the official diagnostic tests. Antibody levels and the number of reactors were lower in feces than in serum in both herds, and no fecal reactors were found in the low-prevalence herd. Therefore, fecal samples showed limited usefulness for antibody detection in goats with this assay, and further studies are needed before any diagnostic application can be considered.

## 1. Introduction

Caprine tuberculosis (cTB) is a chronic zoonotic disease caused by members of the Mycobacterium tuberculosis complex (MTBC), mainly M. caprae and M. bovis [1,2], with M. caprae being the MTBC species most frequently isolated from infected goats in Spain [1]. This disease leads to substantial economic losses [3] and represents a risk for both livestock and human health [4,5], underscoring the importance of effective diagnosis and control of cTB. Spain ranks second (data from 2024) in the European Union for goat population (2,360,950 animals), only behind Greece (2,576,800), highlighting the relevance of cTB surveillance in this species [6].

Ante-mortem diagnosis of cTB traditionally relies on intradermal tests, including single and comparative intradermal tuberculin (SIT and CIT) tests and, in certain epidemiological contexts, the interferon-gamma release assay (IGRA), all of which are based on cellular immune responses [7]. Nevertheless, these methods are not perfect in terms of sensitivity and specificity [8,9,10], prompting the development of complementary approaches that detect antibodies (Ab) against MTBC [11,12,13]. While these tests were initially applied to serum samples [7,9], recent studies have explored alternative, less invasive, and easy-to-collect sampling methods for the detection of specific Ab. Milk has shown potential for this purpose [13], although its use is restricted to lactating females. Consequently, other more universally applicable samples, such as oral fluid or urine, have been investigated in domestic ruminants and wildlife [12,14,15]. These alternatives allow sampling from males and females of all ages, making them particularly useful for herd-level surveillance and wildlife monitoring [12,14,15]. In recent years, several studies have evaluated the performance of different samples for humoral diagnosis of tuberculosis (TB) in ruminants and their potential implementation in the future [12,13,15,16,17,18]. However, previous studies in cattle and goats have demonstrated limited performance of assays based on Ab detection using oral fluid or urine samples for the detection of TB-infected animals [12,15,18]. In this regard, the P22 ELISA is an immunoassay that detects specific Ab against P22, a multiprotein complex immunopurified from the bovine purified protein derivative (bPPD) by affinity chromatography using a monoclonal antibody directed against an epitope shared by MPB70 and MPB83, which are the main components of the complex [19]. This assay has been evaluated in serum, milk, and oral fluid from goats, showing variations in test performance according to the type of sample employed [12,13,16].

To the best of the authors’ knowledge, fecal samples have not been evaluated for Ab-based TB diagnosis in domestic ruminants, even though they have been successfully used for the diagnosis of other pathogens in pigs or wild boar, such as classical swine fever [20] and African swine fever [21], and in domestic ruminants to detect rotavirus [22,23] or *Mycobacterium avium* subsp. *paratuberculosis* (MAP) [24]. While feces have been employed for TB detection in livestock [25,26] and wildlife using Volatile Organic Compounds (VOC) [27,28], the potential of fecal samples for the Ab-based diagnosis of cTB remains unexplored. The detection of specific Ab in feces is biologically plausible. Fecal Ab derives mainly from secretory immunoglobulin A (IgA) produced locally by plasma cells of the intestinal lamina propria and transported across the epithelium, although IgG can also reach the intestinal lumen, mainly in the colon, by a transcytosis process [29,30,31]. Accordingly, pathogen-specific Ab, including IgG and IgA, have been detected in fecal samples from ruminants, including sheep infected with MAP [24,29]. In goats with cTB, exposure of the gut-associated lymphoid tissue to MTBC antigens may occur through swallowed respiratory secretions or through intestinal and mesenteric lymph node involvement, which could stimulate local Ab responses. However, fecal Ab may be present at low concentrations and are exposed to proteolytic degradation in the gastrointestinal tract, so their detection with an assay developed for serum requires specific evaluation. Therefore, given the performance of the P22 ELISA in serum and milk, and the advantages of fecal samples as non-invasive, easy-to-collect samples, we adapted the P22 ELISA to analyze fecal samples from goats. The aim of the present exploratory study was to evaluate the reactivity of the P22 ELISA in fecal samples from goats belonging to two cTB-infected herds, compared with that obtained in serum samples and with the results of the official diagnostic tests, rather than to provide a full diagnostic validation of the assay.

## 2. Materials and Methods

### 2.1. Design of the Study

The study was carried out in two dairy herds of Guadarrama breed goats located in the central region of Spain. Both herds had previously been confirmed as TB-infected using bacteriological culture (*M. bovis*; SB0121). The first herd (*n* = 149; aged between 1 and 6 years) was classified as a high-TB-prevalence herd according to the results of the SIT test (74.5% of reactors) and IGRA (49% of reactors) obtained at the last sampling prior to the present study. The second herd (*n* = 220; aged between 1 and 3 years) was considered a low-TB-prevalence herd due to the results of the SIT test (7.3% of reactors) and IGRA (9.1% of reactors) obtained at the sampling prior to the present study. Moreover, a vaccination routine against MAP was implemented in both TB-infected herds, using the Gudair vaccine (CZ Vaccines, Porriño, Spain) in goats under 6 months of age as part of their vaccination programs. Since vaccination was routinely applied to replacement kids, all the adult goats included in the study were considered vaccinated. Given the high TB-prevalence in the region, the inclusion of a TB-free herd as a control group was not feasible; therefore, the present study evaluated the relative reactivity of fecal samples in infected herds under field conditions, rather than providing a full diagnostic validation of the assay. All goats included in the present study were adult females. The animals were maintained under a semi-extensive management system and received a diet consisting of a mixture of cereals and correctors. Moreover, goats also had daily access to grazing. In order to avoid a more invasive and stressful methodology, those goats that defecated during the sampling (fifty-one from the high-prevalence herd and forty-two belonging to the low-prevalence herd) were included in the present study. This convenience sampling approach may have introduced selection bias, which is addressed in the Discussion. Fecal samples were collected prior to the SIT/CIT test during the daily milking using sterile receptacles in order to avoid possible interference caused by the intradermal tests. For the same reason, blood samples were collected before the intradermal tests in order to obtain plasma for IGRA and serum for P22 ELISA. Goats tested in the present study were not considered experimental animals. All samplings and other procedures were carried out by qualified veterinarians in accordance with Spanish legislation (Royal Decree 2611/1996 and Royal Decree 727/2011). The results for each animal were evaluated individually, with each animal constituting the study unit. Throughout the study, no clinical signs of disease or suffering were observed, and no mortality or adverse events occurred. There were no exclusions and humane endpoints established during the study.

### 2.2. Serum and Fecal Samples Collection

Blood samples were collected from the jugular vein by venipuncture, using plastic serum tubes (BD Vacutainer Becton, Dickinson and Company, Franklin Lakes, NJ, USA). The samples were stored at room temperature (approximately 20–25 °C) for 24 h to allow complete clotting and clot retraction, as routinely performed in our laboratory for serological testing, given that IgG is stable under these conditions, and then centrifuged for 15 min at 650× *g*, after which sera were stored at −20 °C until assaying. Fecal samples were obtained from the same animals during defecation by directly depositing them, using sterile gloves, into sterile receptacles, avoiding contact with the environment and contamination of the fecal samples with environmental mycobacteria. Afterwards, they were frozen until processed. In the laboratory, approximately 1 g of feces was introduced into 30 mL tubes and resuspended in 2 mL of phosphate-buffered saline (PBS) (approximately 1:2, *w*/*v*) by vortexing for 1 min. These mixes were stored at 4 °C for 8 h, after which the samples were centrifuged at 13,000× *g* for 5 min and an aliquot of 500 µL of the supernatant was obtained and stored at −20 °C until analysis.

### 2.3. Diagnostic Tests

#### 2.3.1. SIT/CIT Test

The SIT/CIT tests were carried out by means of the intradermal inoculation of 0.1 mL of bPPD and avian PPD (aPPD) (CZ Vaccines, Porriño, Spain) simultaneously on the left and right sides of the neck, respectively, using a Dermojet syringe (Akra Dermojet, Pau, France). The reactions of the different intradermal tests were interpreted by the same veterinarian in animals from both herds, and the interpretation of the results was carried out as previously described [32]. The SIT and CIT tests were performed according to the protocol published by the European Union Reference Laboratory (EU-RL) for bovine TB (SOP/002/EURL), following the European (Regulation EU 2016/429 and the Commission Delegated Regulation EU 2020/688) and Spanish (Royal Decree 2611/1996) legislation.

#### 2.3.2. IGRA

In order to detect interferon-gamma (IFN-γ) production, blood samples were collected from the jugular vein by venipuncture, using evacuated tubes (BD Vacutainer Becton, Dickinson and Company, Franklin Lakes, USA) with lithium heparin. In the laboratory, these blood samples were stimulated with bPPD and aPPD (CZ Vaccines, Porriño, Spain) at a final concentration of 20 µg/mL each and subsequently processed as described previously [33]. Blood samples were incubated at 37 °C in a humidified atmosphere for 18–20 h. After this time, the samples were centrifuged at 2500 rpm for 15 min, and the supernatant was collected. IFN-γ production in plasma was measured using a commercial kit (Bovigam TB, Thermo Fisher Scientific, Waltham, MA, USA) following the manufacturer’s instructions. Afterwards, the results, expressed as optical density (OD), were interpreted as described elsewhere [32].

### 2.4. P22 ELISA in Serum and Fecal Samples

Serum samples were analyzed by employing a P22 ELISA, as previously described by Infantes-Lorenzo and collaborators [34]. The assay was subsequently adapted for fecal samples as follows. Fecal supernatants, obtained as described in Section 2.2, were tested using the same P22-coated plates, controls, washing steps, substrate, and reading conditions described for serum samples [34]. The optimal dilution of fecal supernatants was established by assessing reactivity across a panel of dilutions (1:1 to 1:16), and a 1:2 dilution was finally selected; diluted samples were added to the plates in duplicate and incubated for 1 h at 37 °C. Bound Ab were detected with a horseradish peroxidase (HRP)-conjugated rabbit anti-sheep IgG (H + L) secondary antibody (Southern Biotech, Birmingham, AL, USA), which cross-reacts with goat IgG; no other detection antibody was used. For fecal samples, this conjugate was titrated from 1:500 to 1:8000, and a working dilution of 1:2000 with an incubation of 30 min at room temperature was selected. As the conjugate recognizes both heavy and light chains, some reactivity against other Ig isotypes sharing light chains (e.g., IgA or IgM) cannot be excluded, although the assay was optimized for IgG detection. Plates were considered valid when the ODs of the positive and negative controls were above 1.5 and below 0.2, respectively.

ELISA results were expressed as an ELISA percentage (E%), which was calculated as:

E% = [mean sample OD/(2 × mean of negative control OD)] × 100

In the case of serum samples, an animal was considered positive to the P22 ELISA when the E% value was ≥150% (standard interpretation; used in low-risk herds) or ≥100% (severe interpretation; used in high-risk herds), as previously described [32,34]. For fecal samples, no established cut-off value exists. Therefore, a receiver operating characteristic (ROC) analysis was performed to evaluate and identify the optimal cut-off point based on the observed P22 ELISA serum results using the ≥100% threshold as the reference classification, including all animals from both herds (*n* = 93). It should be noted that serum P22 ELISA is itself an ancillary Ab-based test and not a definitive reference standard of infection status. The analysis was conducted in R [35] using the “cutpointr” package [36], applying 10,000 bootstrap resamples to estimate performance metrics. The optimal cut-off was selected by maximizing the Youden index (i.e., the sum of sensitivity and specificity); the area under the ROC curve (AUC) and its 95% CI were also estimated.

### 2.5. Statistical Analysis

Quantitative values (E%), observed in fecal and serum samples from the same animals (paired data), were compared separately within each herd using the Wilcoxon signed-rank test. The comparison of the proportions of test reactors to P22 ELISA using different samples in animals from both herds was performed using McNemar’s test. Spearman’s rank correlation coefficient (rs) was used to assess the relationship between E% in fecal and serum samples and between the quantitative results of P22 ELISA (E%) and the values obtained when performing the intradermal test (skin fold thickness in mm) or IGRA (OD). Agreement between tests was measured with the kappa statistic (k), calculated in the reactors to the official diagnostic tests (see Section 3) using the standard interpretation of the official tests and the 150 E% cut-off in serum samples, and interpreted as follows: <0.000 no agreement, 0.000–0.200 slight, 0.201–0.400 fair, 0.401–0.600 moderate, 0.601–0.800 substantial, and 0.801–1.000 almost perfect agreement [37]. The 95% CIs of kappa values were calculated using the asymptotic standard error, and those of Spearman’s coefficients using Fisher’s z-transformation. As a sensitivity analysis, the agreement between the P22 ELISA (serum or feces) and a combined classification based on the official tests (animals positive to the SIT and/or CIT test and/or IGRA, standard interpretation) was also assessed in all the animals from both herds (*n* = 93), and the proportions of goats positive and negative to this combined classification that were also positive and negative, respectively, to the P22 ELISA were calculated. In addition, a ROC analysis of fecal E% values was performed using this combined classification as the reference, independently of serum results (95% CI of the AUC estimated by bootstrap, 2000 resamples), and the number of fecal reactors was recalculated using alternative cut-offs (50 E% and 70 E%) to assess the influence of cut-off uncertainty. Analyses were performed using SPSS Statistics version 29 (IBM, New York, NY, USA) and interpreted using a *p*-value of 0.05 to determine statistical significance. No adjustment for multiple comparisons was applied, given the exploratory nature of the study; therefore, *p*-values should be interpreted with caution. Since the animals belonged to only two herds, observations within each herd may not be fully independent (shared management, exposure, and vaccination history); comparisons were therefore performed separately for each herd whenever possible, and the potential effect of herd clustering, which could not be reliably modeled with only two herds, was considered a limitation of the study. Wilson’s 95% confidence intervals (95% CI) were calculated for the percentage of positive reactors to the different tests using WinPepi version 11.65 [38]. Figures were generated in R version 4.4.1 [35] using the ggplot2 package [39].

## 3. Results

The number and percentage of positive reactors to the different diagnostic tests using different interpretations are summarized in Table 1 (see Supplementary Materials for raw values: Data). Regarding the results obtained using cell-based diagnostic tests, out of the goats belonging to the high-prevalence herd (*n* = 51), 39 [76.5% (95% CI: 63.4–86.0)] animals were positive to SIT test while 8 [15.7% (95% CI: 8.2–28.0)] were positive to CIT test using the standard interpretation of both techniques. Regarding the IGRA results, 22 [43.1% (95% CI: 30.5–56.7)] goats from this herd were positive to this technique in this study using the standard cut-off (Table 1). Out of the goats belonging to the low-prevalence herd (*n* = 42), six [14.3% (95% CI: 6.7–27.8)] animals were positive to SIT test whereas only one [2.4% (95% CI: 0.0–12.3)] to CIT using the standard interpretation of the tests. With regard to the IGRA, five [11.9% (95% CI: 5.2–25.0)] positive animals were observed employing the standard threshold cut-off point for the test interpretation (Table 1).

In the case of fecal samples, the optimal statistical cut-off according to the Youden criterion based on maximizing the sum of sensitivity and specificity was 41.4 E%. Nonetheless, according to the formula used (see Methods), E% values below 50 correspond to sample ODs lower than the mean OD of the negative control; a cut-off within this range would therefore classify as positive samples with a reactivity lower than that of the negative control, which was considered not interpretable. The bootstrapped distribution of optimal cut-offs was variable, although thresholds between 57 E% and 60 E% were among the most frequently selected values (Supplementary Materials Figure S1A,B). Consequently, 60 E% was adopted as the final operational cut-off, selected on the basis of this interpretability constraint and the bootstrap results, rather than as the statistical optimum. Using serum P22 ELISA positivity at the ≥ 100 E% threshold as the reference classification, the ROC analysis yielded an AUC of 0.743 (95% CI: 0.63–0.85), which reflects the ability of the fecal assay to reproduce the serum P22 ELISA classification rather than its ability to detect truly infected animals. This cut-off should therefore be considered preliminary and specific to the present dataset.

**Figure 1:**
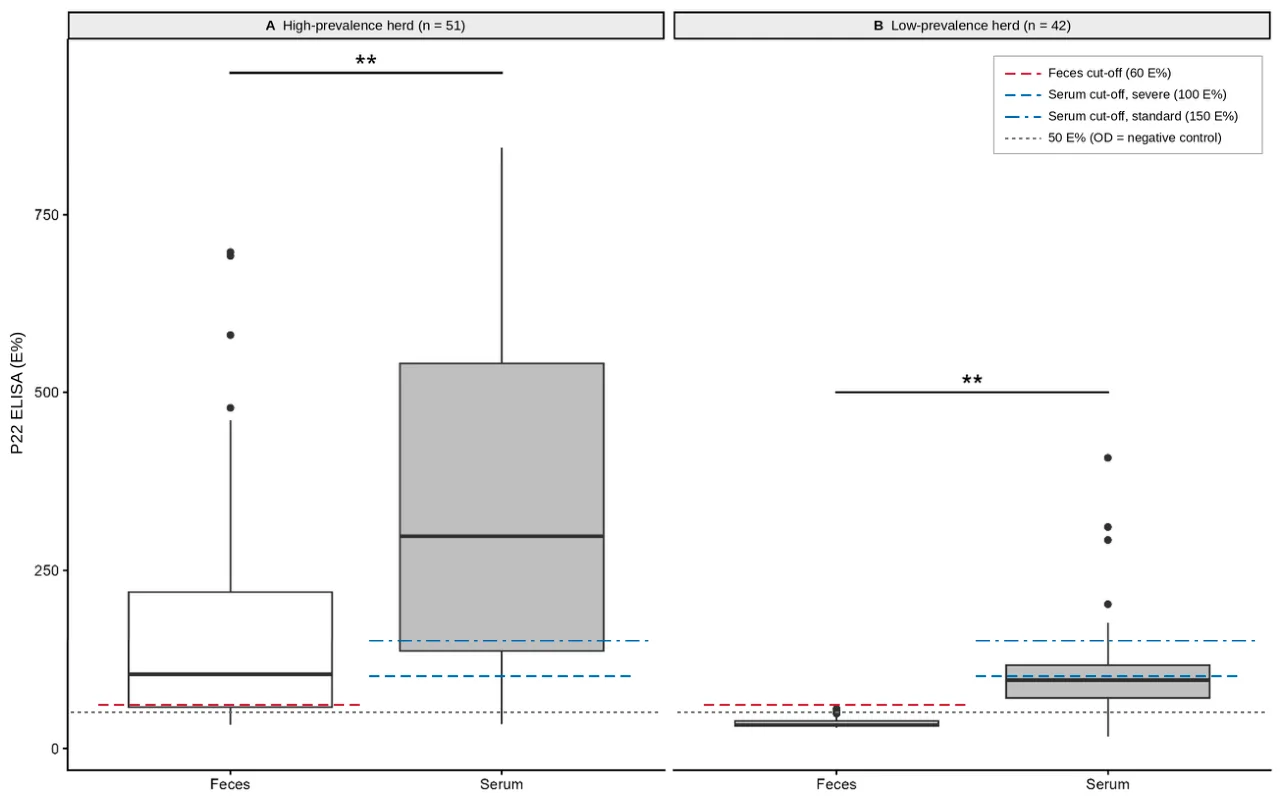
P22 ELISA percentage (E%) in paired fecal (white) and serum (gray) samples from goats of the high-prevalence (*n* = 51) and low-prevalence (*n* = 42) herds. Boxes show the median and interquartile range (IQR), whiskers extend to 1.5 × IQR, and dots represent outliers. Dashed lines indicate the cut-offs applied to fecal (60 E%, preliminary) and serum (100 E% and 150 E%) samples; the dotted line indicates 50 E% (sample OD equal to the mean OD of the negative control). ** Significant differences (Wilcoxon signed-rank test, *p* < 0.001).

In this study, the E% levels observed in serum (median = 297.3 E%) were significantly (*p* < 0.001) higher than in feces (median = 103.4 E%) in animals from the high-prevalence herd (Figure 1). Following the cut-off calculated (60 E%), out of the total 51 animals belonging to the high-prevalence herd, 35 [68.6% (95% CI: 54.9–79.7)] and 44 [86.3% (95% CI: 74.3–93.2)] were positive by the P22 ELISA in fecal and serum samples, respectively, using a cut-off point of 100 E% in serum. Positivity decreased to 37 [72.5% (95% CI: 59.1–82.9)] animals in serum when the cut-off point employed was 150 E%. Thus, a significantly (*p* < 0.05) higher number of reactors to the P22 ELISA was observed using serum (100 E%) compared to fecal samples (60 E%) in the high-prevalence herd. No significant differences (*p* = 0.824) in the number of positive animals to the P22 ELISA were observed between the use of fecal samples and the standard interpretation (150 E%) in serum samples.

With respect to the results obtained in goats from the low-prevalence herd, the E% levels observed in serum (median = 95.0 E%) were significantly (*p* < 0.001) higher than in feces (median = 32.8 E%) (Figure 1). Moreover, none of the animals in this herd was positive to the P22 ELISA using fecal samples (60 E%) compared to 19 [45.2% (95% CI: 31.2–60.0)] and 5 [11.9% (95% CI: 5.2–25.0)] goats that were positive in serum samples using the 100 E% and 150 E% cut-off points, respectively (Table 1). Similar to the results observed in the high-prevalence herd, the number of positive animals to P22 ELISA in serum samples (cut-off point of 100 E%) was significantly (*p* < 0.001) higher than that observed using fecal samples in animals belonging to the low-prevalence herd, but the differences in positivity observed between both samples were not significant (*p* = 0.063) when employing a cut-off of 150 E% in serum samples.

**Figure 2:**
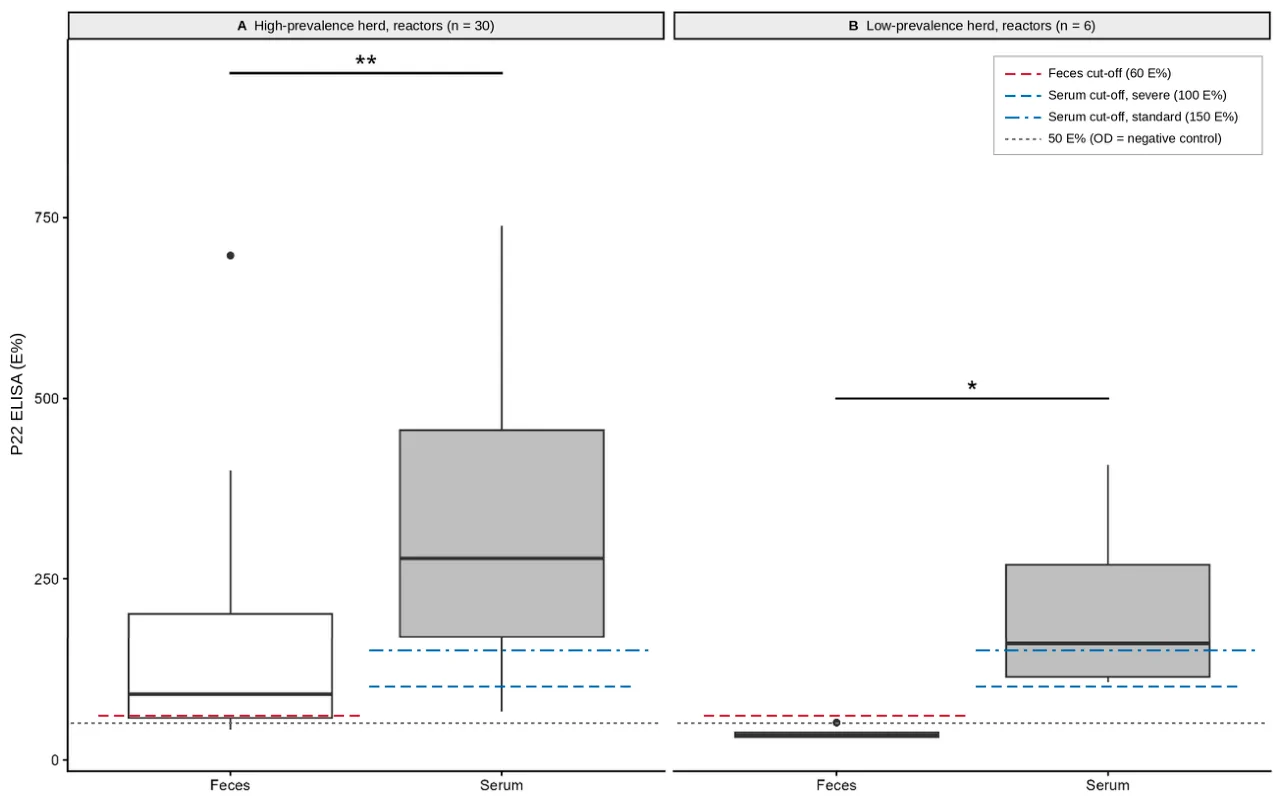
P22 ELISA percentage (E%) in paired fecal (white) and serum (gray) samples from reactors to the official diagnostic tests (SIT and/or CIT test and/or IGRA) in the high-prevalence (*n* = 30) and low-prevalence (*n* = 6) herds. Boxes show the median and IQR, whiskers extend to 1.5 × IQR and dots represent outliers. Dashed and dotted lines as in Figure 1. Significant differences (Wilcoxon signed-rank test): *p* < 0.05, * *p* < 0.001.

There was no correlation between the E% levels observed in serum and fecal samples from those animals of the high- (r_s_ = −0.043; 95% CI: −0.315 to 0.235) and low-prevalence herds (r_s_ = 0.035; 95% CI: −0.272 to 0.335). The reactors to official diagnostic tests (*n* = 36; goats positive to the CIT test, standard interpretation, and/or to IGRA, severe interpretation) had a significantly higher E% (*p* < 0.001) when using serum (median = 255.5 E%) than when using fecal samples (median = 70.7 E%). In the high-prevalence herd, the E% levels of reactors to official diagnostic tests (*n* = 30) were significantly higher (*p* < 0.001) in serum (median = 278.4 E%) compared to fecal samples (median = 90.1 E%). Equally, the results of reactors (*n* = 6) to official techniques from the low-prevalence herd showed significantly higher (*p* < 0.05) E% levels in serum (median = 159.6 E%) compared to fecal samples (median = 33.8 E%) (Figure 2). No correlation (r_s_ = 0.004; 95% CI: −0.325 to 0.332) was observed between the E% levels in serum and fecal samples from reactors to the official diagnostic tests. Moreover, a weak positive correlation, not statistically significant (rs = 0.326; 95% CI: −0.003 to 0.591; *p* = 0.052), was observed between the E% levels in fecal samples and the increase in skin fold thickness (mm) at the bPPD inoculation site in these animals, whereas the correlations between fecal E% levels and the quantitative results of the CIT test (difference between bovine and avian reactions, mm; rs = 0.119; 95% CI: −0.218 to 0.431) and IGRA (OD; rs = −0.056; 95% CI: −0.377 to 0.278) were negligible. Moreover, a slight agreement (kappa value) was observed between the reactivity to SIT/CIT tests and P22 ELISA in the reactors to the official diagnostic tools in general terms. A slight agreement was observed between the SIT test and the P22 ELISA in fecal (k = 0.195; 95% CI: −0.071 to 0.461) or serum samples (k = 0.008; 95% CI: −0.312 to 0.329), and between the CIT test and the P22 ELISA using feces (k = 0.027; 95% CI: −0.247 to 0.301) or serum samples (k = 0.136; 95% CI: −0.047 to 0.320). No agreement was observed between IGRA and the P22 ELISA using fecal samples (k = −0.029; 95% CI: −0.318 to 0.261), whereas a slight agreement was found between IGRA and the P22 ELISA using serum samples (k = 0.071; 95% CI: −0.265 to 0.408).

In the sensitivity analysis, 50 goats were positive to the combined classification based on the official tests (SIT and/or CIT test and/or IGRA, standard interpretation; 42 in the high- and 8 in the low-prevalence herd). Of these, 29 [58.0% (95% CI: 44.2–70.6)] were positive to the P22 ELISA in fecal samples (60 E%), compared with 42 [84.0% (95% CI: 71.5–91.7)] and 35 [70.0% (95% CI: 56.2–80.9)] in serum samples using the 100 E% and 150 E% cut-offs, respectively. Among the 43 goats negative to the combined classification, 37 [86.0% (95% CI: 72.7–93.4)], 22 [51.2% (95% CI: 36.8–65.4)], and 36 [83.7% (95% CI: 70.0–91.9)] were negative to the P22 ELISA in feces and in serum at 100 E% and 150 E%, respectively. The overall agreement with the combined classification was moderate for fecal samples (k = 0.430; 95% CI: 0.258–0.602) and fair to moderate for serum samples (k = 0.359; 95% CI: 0.175–0.543 and k = 0.530; 95% CI: 0.361–0.699 using 100 E% and 150 E%, respectively). However, these pooled estimates were largely driven by the differences between herds: within the high-prevalence herd, the agreement between the combined classification and fecal P22 ELISA was negligible (k = 0.018; 95% CI: −0.241 to 0.277), and none of the eight test-positive goats from the low-prevalence herd was positive using fecal samples. The ROC analysis of fecal E% values using the combined classification as the reference, independently of serum results, yielded an AUC of 0.803 (95% CI: 0.700–0.890) and identified the same statistical optimum (41.4 E%) as the analysis based on serum P22 ELISA. Regarding cut-off uncertainty, in the high-prevalence herd 44 [86.3% (95% CI: 74.3–93.2)], 35 [68.6% (95% CI: 54.9–79.7)], and 31 [60.8% (95% CI: 47.1–73.0)] goats were positive in fecal samples using 50 E%, 60 E%, and 70 E% as cut-off, respectively, whereas in the low-prevalence herd only 2 goats [4.8% (95% CI: 1.3–15.8)] exceeded 50 E% and none exceeded 60 E% or 70 E%. These estimates reflect concordance between imperfect tests and should not be interpreted as measures of diagnostic sensitivity or specificity.

## 4. Discussion

In the present study, an in-house ELISA, previously evaluated for detecting Ab against MTBC members in serum and milk samples in goats [13,16,17,34], was adapted for the first time for its use with caprine fecal samples. Overall, the assay showed limited reactivity in fecal samples, whereas serum consistently provided higher reactivity in both the high- and low-prevalence herds. These findings aligned with previous studies in goats and other domestic ruminants, where antibody-based assays generally exhibited superior performance in serum samples compared to other alternatives, including oral fluid or milk [12,13]. In the high-prevalence herd, the number of positive animals to P22 ELISA obtained with fecal samples was slightly higher than that previously reported for oral fluid [12]. In our study, no positive results were obtained in fecal samples from the low-prevalence herd, suggesting that, under the conditions of this study, fecal samples may have limited usefulness for Ab detection in herds with a low number of infected animals; however, only one herd of each category was evaluated, and this observation should be confirmed in a larger number of herds. An alternative explanation may be differences in the stage or intensity of infection between the two herds; however, this hypothesis could not be evaluated in the present study because individual infection status and time since infection were not established. The lower reactivity observed in fecal samples compared with serum may be explained by several non-mutually exclusive factors. First, the predominant Ig differs between sample types: IgG predominates in serum, whereas IgA is the most prevalent isotype at mucosal surfaces in adult ruminants [29,30,31]. Since the assay uses an anti-IgG (H+L) conjugate optimized for serum IgG, fecal Ab of other isotypes, particularly IgA, are likely to be detected only partially (through light-chain recognition); this hypothesis could not be tested, as isotype-specific conjugates were not used. Second, the concentration of IgG in feces is expected to be much lower than in serum [30], and fecal Ab are exposed to proteolytic degradation by host and microbial enzymes during intestinal transit, which may reduce their stability and integrity. Third, matrix effects, the presence of interfering substances in fecal material, the dilution of the sample, and the efficiency of the extraction procedure may also have reduced the signal. These factors should be regarded as possible explanations rather than demonstrated mechanisms, and further studies evaluating isotype-specific detection, the use of protease inhibitors or alternative extraction protocols would be needed. By contrast, serum and milk samples have shown better diagnostic performance and may be more suitable for large-scale testing in goats [16]. Moreover, there was no correlation between E% levels in serum and fecal samples in both herds, including reactors to official diagnostic tools. These findings differ from previous studies in sheep infected with nematodes reporting significant positive correlation between plasma and fecal Ab levels for IgG and IgA. In addition, the agreement observed in the present study between official diagnostic tools (intradermal tests and IGRA) and P22 ELISA, regardless of the sample used (serum or feces), was slight in general terms, probably because both are techniques based on different immune responses (cellular or humoral). Cell-mediated responses usually predominate in the early stages of infection, whereas humoral responses tend to increase as the disease progresses, so both types of tests may identify partially different subsets of infected animals [8,10]. In the case of fecal samples, the lack of agreement with IGRA and the slight agreement with the intradermal tests probably reflect a combination of these different immune response profiles, possible differences in disease stage among animals and the limitations of Ab detection in feces discussed above. However, in the absence of post-mortem or culture confirmation, discordant results cannot be classified as false-positive, false-negative, or stage-dependent responses, and the present data do not support the use of fecal P22 ELISA to reliably identify cTB-infected goats. The sensitivity analysis based on the combined classification of all official tests supported these findings: the apparently moderate pooled agreement was mainly explained by the differences between herds, fecal P22 ELISA did not detect any of the test-positive goats from the low-prevalence herd, and the ROC analysis performed independently of serum results identified the same statistical optimum as that based on serum P22 ELISA, indicating that the fecal cut-off was not merely an artifact of using serum as the reference, although it should still be regarded as preliminary. These results align with a previous large-scale study where a slight agreement between the P22 ELISA (using serum and milk samples) and intradermal tests was observed [16]. Therefore, this fact reflects the potential implementation of P22 ELISA in serum as a complementary tool for cTB diagnosis in an eradication program scenario. Overall, the results of the present study indicate that the use of fecal samples for stand-alone Ab-based TB diagnosis remains limited and far from routine application in livestock. It should be noted that fecal Ab production is related to mucosal immunity, which develops through local antigen stimulation, making this stimulation of paramount importance if high Ab levels are to be attained in fecal samples. This could be one explanation for the differences observed in the present study between fecal and serum samples. Although the present study was carried out in goats, the P22 ELISA performance could be similar or even lower in cattle from countries with an eradication program for bovine TB, one of which is Spain, as the high diagnostic pressure resulting from the eradication process conducted over decades significantly reduces the likelihood of animals reaching advanced stages of the disease and, consequently, presenting high Ab titers. However, additional studies would be necessary in cattle in order to confirm this hypothesis and in other non-domestic ruminants, where control programs are not applied.

The present study has several limitations that should be considered when interpreting the results. First, no TB-free herd could be included as a control group, which prevents a robust estimation of specificity and of a cut-off value independent of serum results. Second, no post-mortem data (lesions and/or bacteriological culture) or molecular confirmation (e.g., PCR) were available at the individual level (the absence of an eradication program at national level makes it difficult to cull animals under field conditions, particularly those that are positive only by experimental tests); without such confirmation, discordant results between official tests, serum P22 ELISA, and fecal P22 ELISA cannot be definitively classified as false-positive, false-negative, or stage-dependent immune responses. Third, herd classification (high or low prevalence) was based on previous SIT and IGRA results, which have imperfect sensitivity and specificity. In any case, these are official tests with a high sensitivity at the herd level [10], which allow the herd status to be reliably determined. Serum P22 ELISA, used as the reference for the fecal cut-off, is also an ancillary Ab-based test rather than a definitive reference standard. Consequently, the proportions of reactors and the agreement estimates reported here represent apparent reactivity and agreement relative to other imperfect tests, not true diagnostic sensitivity and specificity, and the 60 E% cut-off should be considered preliminary and specific to this dataset, as also illustrated by the variability of the bootstrapped cut-off distribution (Supplementary Materials Figure S1). Fourth, only goats that defecated during sampling were included (51 out of 149 and 42 out of 220 animals), which may have introduced selection bias. Although the proportions of SIT and IGRA reactors among the sampled goats (76.5% and 43.1% in the high-prevalence herd; 14.3% and 11.9% in the low-prevalence herd) were broadly similar to those observed in the whole herds at the previous sampling (74.5% and 49.0%; 7.3% and 9.1%, respectively), individual data on the age or diagnostic history of non-sampled animals were not available for a formal comparison. Fifth, all the animals were adult females of a single breed kept under a semi-extensive system, so the results may not be directly generalizable to males, young animals, or other management systems. Sixth, both herds were vaccinated against MAP, which can interfere with TB diagnostic tests in goats, including the SIT test, IGRA, and serological assays, although this interference decreases over time after vaccination [9]. Since vaccination was applied to kids under 6 months of age and all study animals were adults, this effect is expected to be limited, but a contribution of vaccination to the reactivity observed in the official tests and in serum P22 ELISA cannot be excluded, whereas its potential influence on fecal Ab remains unknown. The results should therefore be regarded as exploratory.

## 5. Conclusions

In conclusion, under the field conditions of the present study, fecal P22 ELISA showed limited value for the humoral detection of cTB, particularly in the low-prevalence herd. Ab levels and the number of reactors were significantly lower in fecal than in serum samples, indicating that serum remained the more suitable sample for Ab-based testing with this assay. Fecal samples may have limited exploratory value in heavily infected herds, but this possibility, as well as their usefulness in other epidemiological settings, requires further investigation. Further validation including TB-free controls and animals with post-mortem confirmed infection status is required before any diagnostic application can be recommended.

## Figures and Tables

**Table 1:** Number and percentage of reactors to the single and comparative intradermal tuberculin (SIT and CIT) tests, interferon-gamma release assay (IGRA), and P22 ELISA in serum and fecal samples.

Herd	*n*	SIT Test ^a^		CIT Test ^b^		IGRA 0.05 ^c^	IGRA 0.1 ^c^	P22 ELISA in Serum 100 ^d^	P22 ELISA in Serum 150 ^d^	P22 ELISA in Feces 60 ^d^
		Standard	Severe	Standard	Severe					
High prevalence	51	39, 76.5 (63.4–86.0) ^e^	86.3 (74.3–93.2)	8, 15.7 (8.2–28.0)	30, 58.8 (45.2–71.3)	28, 54.9 (41.4–67.7)	22, 43.1 (30.5–56.7)	86.3 (74.3–93.2)	37, 72.5 (59.1–82.9)	35, 68.6 (54.9–79.7)
Low prevalence	42	6, 14.3 (6.7–27.8)	8, 19.0 (9.9–33.3)	1, 2.4 (0.0–12.3)	6, 14.3 (6.7–27.8)	6, 14.3 (6.7–27.8)	5, 11.9 (5.2–25.0)	19, 45.2 (31.2–60.0)	5, 11.9 (5.2–25.0)	0, 0.0 (0.0–8.4)

^a^ An animal was considered a positive reactor to the single intradermal tuberculin (SIT) test when there was an increase ≥4 mm (standard interpretation) or >2 mm (severe interpretation) in the skin fold thickness and/or the presence of clinical signs were observed. ^b^ An animal was considered a positive reactor to the comparative intradermal tuberculin (CIT) test when the bovine reaction was greater than the avian reaction in more than 4 mm (standard interpretation) or the bovine reaction was ≥3 mm and greater than the avian reaction (severe interpretation); and/or the presence of clinical signs at the bovine PPD inoculation site. ^c^ An animal was considered positive to the IGRA if the optical density (OD) of a sample stimulated with bovine PPD minus the OD of PBS was greater than 0.1 (standard interpretation) or 0.05 (severe interpretation) and greater than the OD of the sample stimulated with avian PPD. ^d^ An animal was considered positive to P22 ELISA in serum samples when the E% was ≥150 (standard interpretation) or ≥100 (severe interpretation) and in fecal samples when the E% value was ≥60 (preliminary cut-off; see text). ^e^ Wilson’s 95% confidence intervals.

## Data Availability

The original contributions presented in this study are included in the article and Supplementary Materials. Further inquiries can be directed to the corresponding author.

## Supplementary Materials

The following supporting information can be downloaded at: https://www.mdpi.com/article/10.3390/vetsci13101083/s1, Figure S1A,B: Cut-off estimation for P22 ELISA on fecal samples (considering P22 ELISA on serum samples as reference). A. Bootstrapped distribution of optimal cut-off points. B. Sum of the specificity and sensitivity of possible optimal cut-off points based on the bootstrapped distribution. Gray area represents the 95% confidence interval.

## References

[1] Rodríguez S., Bezos J., Romero B., de Juan L., Álvarez J., Castellanos E., Moya N., Lozano F., Javed M.T., Sáez-Llorente J.L. (2011). *Mycobacterium caprae* Infection in Livestock and Wildlife, Spain. Emerg. Infect. Dis..

[2] Cadmus S.I., Adesokan H.K., Jenkins A.O., van Soolingen D. (2009). *Mycobacterium bovis* and *M. tuberculosis* in goats, Nigeria. Emerg. Infect. Dis..

[3] Daniel R., Evans H., Rolfe S., de la Rua-Domenech R., Crawshaw T., Higgins R.J., Schock A., Clifton-Hadley R. (2009). Outbreak of tuberculosis caused by *Mycobacterium bovis* in golden Guernsey goats in Great Britain. Vet. Rec..

[4] Napp S., Allepuz A., Mercader I., Nofrarías M., López-Soria S., Domingo M., Romero B., Bezos J., Pérez de Val B. (2013). Evidence of goats acting as domestic reservoirs of bovine tuberculosis. Vet. Rec..

[5] Martínez-Lirola M., Herranz M., Buenestado Serrano S., Rodríguez-Grande C., Dominguez Inarra E., Garrido-Cárdenas J.A., Correa Ruiz A.M., Bermúdez M.P., Causse Del Río M., González Galán V. (2023). A One Health approach revealed the long-term role of *Mycobacterium caprae* as the hidden cause of human tuberculosis in a region of Spain, 2003 to 2022. Euro Surveill..

[6] Eurostat (2025). Goats Population—Anual Data. https://ec.europa.eu/eurostat/databrowser/view/APRO_MT_LSGOAT/default/table?lang=en.

[7] Roy A., Infantes-Lorenzo J.A., de la Cruz M.L., Dominguez L., Alvarez J., Bezos J. (2020). Accuracy of tuberculosis diagnostic tests in small ruminants: A systematic review and meta-analysis. Prev. Vet. Med..

[8] Bezos J., Álvarez J., Romero B., Aranaz A., de Juan L. (2012). Tuberculosis in goats: Assessment of current in vivo cell-mediated and antibody-based diagnostic assays. Vet. J..

[9] Roy Á., Infantes-Lorenzo J.A., Blázquez J.C., Venteo Á., Mayoral F.J., Domínguez M., Moreno I., Romero B., de Juan L., Grau A. (2018). Temporal analysis of the interference caused by paratuberculosis vaccination on the tuberculosis diagnostic tests in goats. Prev. Vet. Med..

[10] Bezos J., Casal C., Romero B., Schroeder B., Hardegger R., Raeber A.J., López L., Rueda P., Domínguez L. (2014). Current *ante-mortem* techniques for diagnosis of bovine tuberculosis. Res. Vet. Sci..

[11] Jiménez-Martín D., García-Bocanegra I., Risalde M.A., Napp S., Domínguez M., Romero B., Moreno I., Martínez R., Cano-Terriza D. (2024). *Mycobacterium tuberculosis* complex in domestic goats in southern Spain. Prev. Vet. Med..

[12] Ortega J., Infantes-Lorenzo J.A., Bezos J., Roy A., de Juan L., Romero B., Moreno I., Gómez-Buendía A., Agulló-Ros I., Domínguez L. (2021). Evaluation of P22 ELISA for the detection of *Mycobacterium bovis*-specific antibody in the Oral fluid of goats. Front. Vet. Sci..

[13] Ortega J., Infantes-Lorenzo J.A., Roy A., de Juan L., Romero B., Moreno I., Domínguez M., Domínguez L., Bezos J. (2022). Factors affecting the performance of P22 ELISA for the diagnosis of caprine tuberculosis in milk samples. Res. Vet. Sci..

[14] Barasona J.A., Barroso-Arévalo S., Rivera B., Gortázar C., Sánchez-Vizcaíno J.M. (2020). Detection of antibodies against *Mycobacterium bovis* in oral fluid from Eurasian wild boar. Pathogens.

[15] Lyashchenko K.P., Sikar-Gang A., Sridhara A.A., Johnathan-Lee A., Elahi R., Greenwald R., Lambotte P., Esfandiari J., Roos E.O., Kerr T.J. (2021). Use of blood matrices and alternative biological fluids for antibody detection in animal tuberculosis. Vet. Immunol. Immunopathol..

[16] Velasco C., Ortega J., Alvarez J., Infantes Lorenzo J.A., Moreno J.C., Sanz C., Romero B., de Juan L., Dominguez L., Dominguez M. (2025). Field evaluation of the P22 ELISA for diagnosis of caprine tuberculosis in an endemic area. Front. Vet. Sci..

[17] Bezos J., Roy A., Infantes-Lorenzo J.A., González I., Venteo Á., Romero B., Grau A., Mínguez O., Domínguez L., de Juan L. (2018). The use of serological tests in combination with the intradermal tuberculin test maximizes the detection of tuberculosis infected goats. Vet. Immunol. Immunopathol..

[18] Kelley H.V., Waibel S.M., Sidiki S., Tomatis-Souverbielle C., Scordo J.M., Hunt W.G., Barr N., Smith R., Silwani S.N., Averill J.J. (2020). Accuracy of Two Point-of-Care Tests for Rapid Diagnosis of Bovine Tuberculosis at Animal Level using Non-Invasive Specimens. Sci. Rep..

[19] Infantes-Lorenzo J.A., Moreno I., Risalde M.A., Roy A., Villar M., Romero B., Ibarrola N., de la Fuente J., Puentes E., de Juan L. (2017). Proteomic characterisation of bovine and avian purified protein derivatives and identification of specific antigens for serodiagnosis of bovine tuberculosis. Clin. Proteom..

[20] Seo S.W., Sunwoo S.Y., Hyun B.H., Lyoo Y.S. (2012). Detection of antibodies against classical swine fever virus in fecal samples from wild boar. Vet. Microbiol..

[21] Nieto-Pelegrín E., Rivera-Arroyo B., Sánchez-Vizcaíno J.M. (2015). First detection of antibodies against African swine fever virus in faeces samples. Transbound. Emerg. Dis..

[22] Parreño V., Marcoppido G., Vega C., Garaicoechea L., Rodriguez D., Saif L., Fernández F. (2010). Milk supplemented with immune colostrum: Protection against rotavirus diarrhea and modulatory effect on the systemic and mucosal antibody responses in calves experimentally challenged with bovine rotavirus. Vet. Immunol. Immunopathol..

[23] Huang Y., Li Z., Fu Y., Wang S.Q., Kang M., Meng R. (2025). Diagnosis of bovine rotavirus: An overview of currently available methods. Front. Microbiol..

[24] Begg D.J., de Silva K., Plain K.M., Purdie A.C., Dhand N., Whittington R.J. (2015). Specific faecal antibody responses in sheep infected with *Mycobacterium avium* subspecies *paratuberculosis*. Vet. Immunol. Immunopathol..

[25] Rodríguez-Hernández P., Cardador M.J., Ríos-Reina R., Sánchez-Carvajal J.M., Galán-Relaño Á., Jurado-Martos F., Luque I., Arce L., Gómez-Laguna J., Rodríguez-Estévez V. (2023). Detection of *Mycobacterium tuberculosis* complex field infections in cattle using fecal volatile organic compound analysis through gas chromatography-ion mobility spectrometry combined with chemometrics. Microbiol. Spectr..

[26] Ellis C.K., Rice S., Maurer D., Stahl R., Waters W.R., Palmer M.V., Nol P., Rhyan J.C., VerCauteren K.C., Koziel J.A. (2017). Use of fecal volatile organic compound analysis to discriminate between non-vaccinated and BCG-Vaccinated cattle prior to and after *Mycobacterium bovis* challenge. PLoS ONE.

[27] de Jesús Beleño-Sáenz K., Cáceres-Tarazona J.M., Nol P., Jaimes-Mogollón A.L., Gualdrón-Guerrero O.E., Durán-Acevedo C.M., Barasona J.A., Vicente J., Torres M.J., Welearegay T.G. (2021). Non-Invasive Method to Detect Infection with *Mycobacterium tuberculosis* Complex in Wild Boar by Measurement of Volatile Organic Compounds Obtained from Feces with an Electronic Nose System. Sensors.

[28] Stahl R.S., Ellis C.K., Nol P., Waters W.R., Palmer M., VerCauteren K.C. (2015). Fecal Volatile Organic Ccompound Profiles from White-Tailed Deer (*Odocoileus virginianus*) as Indicators of *Mycobacterium bovis* Exposure or *Mycobacterium bovis* Bacille Calmette-Guerin (BCG) Vaccination. PLoS ONE.

[29] Cooke A.S., Watt K.A., Morgan E.R., Dungait J.A.J. (2019). The latest FAD—Faecal antibody detection in cattle. Protocol and results from three UK beef farms naturally infected with gastrointestinal nematodes. Parasitology.

[30] Watt K.A., Nussey D.H., Maclellan R., Pilkington J.G., McNeilly T.N. (2015). Fecal antibody levels as a noninvasive method for measuring immunity to gastrointestinal nematodes in ecological studies. Ecol. Evol..

[31] Suzuki Y., Oishi M., Hirota S., Hayashi H., Haga S., Koike S., Kobayashi Y. (2025). Developmental changes in the capacity for mucosal immunoglobulin production and secretion in the intestines of growing calves. Vet. Res..

[32] Ortega J., Roy Á., Álvarez J., Sánchez-Cesteros J., Romero B., Infantes-Lorenzo J.A., Sáez J.L., López M., Domínguez L., de Juan L. (2021). Effect of the inoculation site of bovine and avian purified protein derivatives (PPDs) on the performance of the intradermal tuberculin test in goats from tuberculosis free and infected herds. Front. Vet. Sci..

[33] Bezos J., Alvarez J., de Juan L., Romero B., Rodríguez S., Castellanos E., Saéz-Llorente J.L., Mateos A., Domínguez L., Aranaz A. (2011). Factors influencing the performance of an interferon-gamma assay for the diagnosis of tuberculosis in goats. Vet. J..

[34] Infantes-Lorenzo J.A., Moreno I., Roy A., Risalde M.A., Balseiro A., de Juan L., Romero B., Bezos J., Puentes E., Åkerstedt J. (2019). Specificity of serological test for detection of tuberculosis in cattle, goats, sheep and pigs under different epidemiological situations. BMC Vet. Res..

[35] R Core Team (2025). R: A Language and Environment for Statistical Computing.

[36] Thiele C., Hirschfeld G. (2021). cutpointr: Improved Estimation and Validation of Optimal Cutpoints in R. J. Stat. Soft..

[37] Landis J.R., Koch G.G. (1977). The measurement of observer agreement for categorical data. Biometrics.

[38] Abramson J.H. (2011). WINPEPI updated: Computer programs for epidemiologists, and their teaching potential. Epidemiol. Perspect. Innov..

[39] Wickham H. (2016). ggplot2, Elegant Graphics for Data Analysis.

