# Supplementary material for: Exploratory Evaluation of the P22 ELISA for Antibody Detection in Fecal Samples from Tuberculosis-Infected Goat Herds
Source: Vet Sci. 2026 Oct 13;13(10):1083. doi: 10.3390/vetsci13101083 (PMC13709756; doi:10.3390/vetsci13101083)

**Supplementary Material**.

**Supplementary Figure S1**

Cut-off estimation for P22 ELISA on fecal samples (considering P22 ELISA on serum samples as reference). A. Bootstrapped distribution of optimal cut-off points. B. Sum of the specificity and sensitivity of possible optimal cut-off points based on the bootstrapped distribution. Gray area represents the 95% confidence interval.

**A**

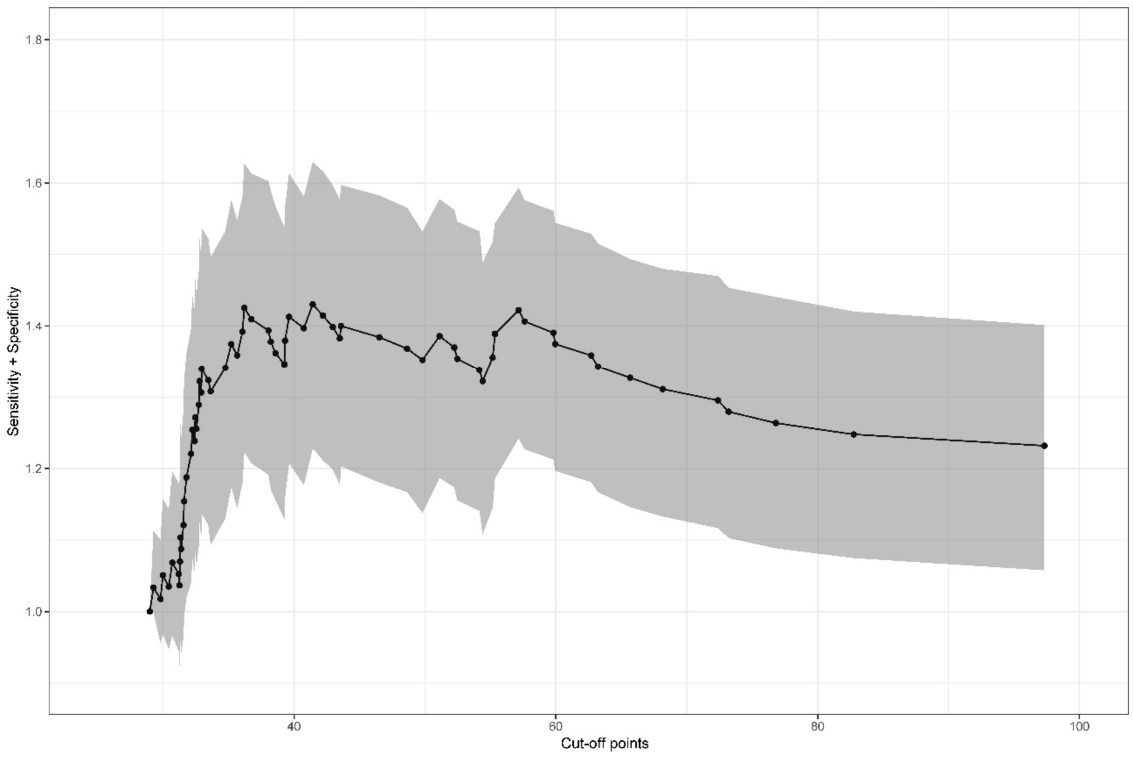

**B**

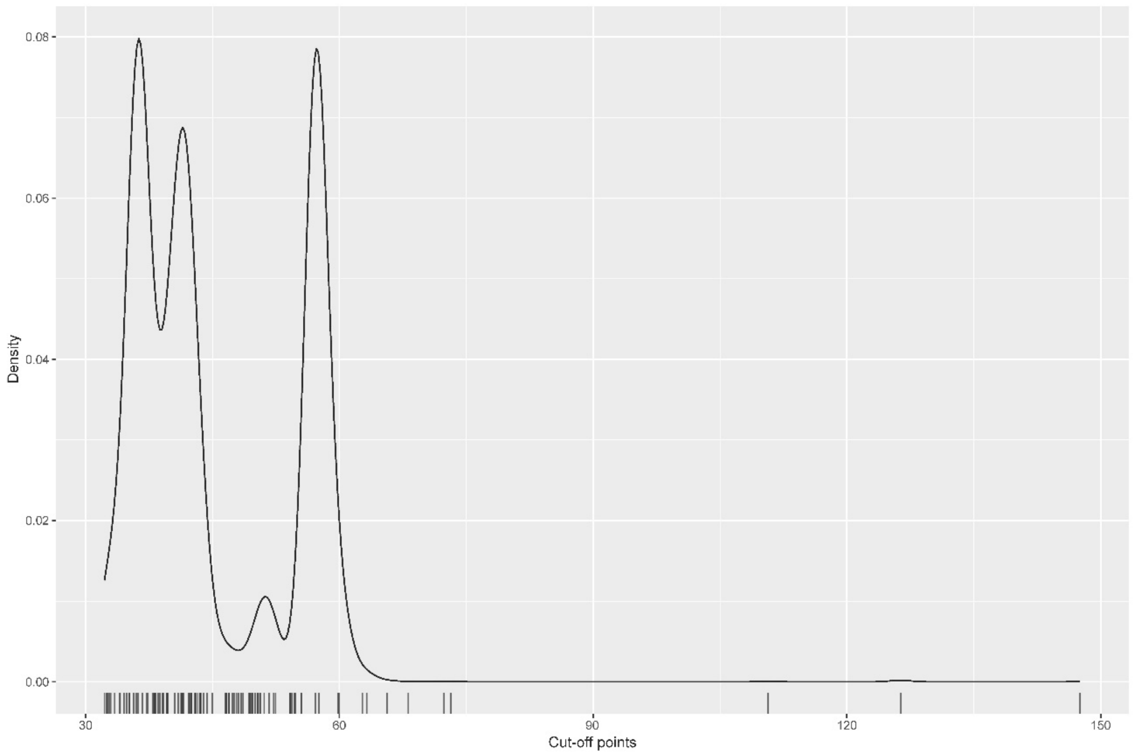

Supplement: Supplementary file 1 [file vetsci-13-01083-s001.zip › vetsci-4588329-supplementary.pdf]
